# Association between Life’s Essential 8 and psoriasis in US adults: a cross-sectional study

**DOI:** 10.3389/fmed.2024.1445288

**Published:** 2024-10-10

**Authors:** Junjie Zhang, Ci Ren, Zihan Qin, Ling Zhu, Zhoufeng Jin, Yuanyuan Yan, Xinghe Pan, Lan Luan

**Affiliations:** ^1^Department of Pathology, Central Hospital Affiliated to Shenyang Medical College, Shenyang, China; ^2^Department of Dermatological Surgery, Shenyang Seventh People’s Hospital, Shenyang, China; ^3^Department of Gastroenterology, Beijing Friendship Hospital, Capital Medical University, Beijing, China; ^4^State Key Laboratory of Digestive Health, Beijing, China; ^5^National Clinical Research Center for Digestive Diseases, Beijing, China; ^6^Medical Technology Department, Sichuan Nursing Vocational College, Chengdu, China; ^7^Department of Plastic Surgery, Shenyang Mingliu Plastic Surgery and Aesthetics Hospital, Shenyang, China; ^8^Affiliated Hospital of Tianjin Academy of Traditional Chinese Medicine, Tianjin, China; ^9^Department of General Surgery, Central Hospital Affiliated to Shenyang Medical College, Shenyang, China

**Keywords:** psoriasis, Life’s Essential 8, cardiovascular health, NHANES, risk factors

## Abstract

**Background:**

Psoriasis is closely associated with cardiovascular disease (CVD). However, the current evidence on the correlation between Life’s Essential 8 and Psoriasis is insufficient. Our aim was to investigate the relationship between Life’s Essential 8 (LE8), a measure of cardiovascular health (CVH), and psoriasis.

**Objective:**

This study aimed to clarify the impact of Life’s Essential 8 on Psoriasis and explore its implications.

**Methods:**

This population-based cross-sectional study included 9,876 US adults aged 20 to 59 years from the National Health and Nutrition Examination Survey (NHANES) 2003–2006 and 2009–2014 cycles. The LE8 score comprises 8 metrics and was categorized into low, moderate, and high CVH. Logistic regression and restricted cubic splines (RCS) were used to assess the association between LE8 score and psoriasis.

**Results:**

Among the 9,876 participants, those with moderate and high CVH had higher risks of psoriasis compared to low CVH. Additionally, every 10-point increase in the LE8 score was associated with a 10% reduced risk of psoriasis. Interaction was observed between gender, age, education level, race/ethnicity, marital status, and PIR.

**Conclusion:**

LE8 and its subscale scores were strongly negatively related to the risk of psoriasis. Encouraging optimal CVH levels may be advantageous in reducing the incidence of psoriasis.

## 1 Introduction

Psoriasis, a chronic immune-mediated systemic disease, is characterized by red patches covered with silvery scales, often causing itching, pain, and in severe cases, joint involvement. It is associated with cardiovascular diseases, diabetes, and metabolic syndrome, significantly impacting patients’ quality of life. Early identification and treatment of cardiovascular complications are crucial for improving patient outcomes (1). A recent study utilizing NHANES data from 2005–2014 first applied the Life’s Essential 8 (LE8) metric to the psoriasis population and found that individuals with psoriasis had a higher prevalence of poor cardiovascular health (CVH), as indicated by lower LE8 scores, compared to those without psoriasis (2). This pioneering work highlighted the utility of LE8 in this high-risk population. However, a comprehensive dose-response analysis and investigation of the independent contributions of individual LE8 components to psoriasis risk remain unexplored.

The Life’s Essential 8 (LE8), an algorithm developed by the American Heart Association, assesses cardiovascular health through diet, physical activity, smoking habits, sleep patterns, body mass index, lipid status, blood glucose levels, and blood pressure. Understanding the relationship between LE8 and psoriasis is critical in healthcare and public health (3). This study aims to quantify the association between cardiovascular health levels, as measured by LE8 scores, and the prevalence of psoriasis among US adults.

Existing research indicates that psoriasis patients with cardiovascular risk factors have a higher prevalence of traditional cardiovascular risk factors, such as type 2 diabetes, hypertension, lipid abnormalities, and obesity (4), with obesity being an independent factor in psoriasis (4). The systemic inflammation caused by psoriasis is a key factor in increased cardiovascular risk (5), making cardiovascular disease a leading cause of mortality among psoriasis patients (6). Furthermore, psoriasis patients are at an increased risk of subclinical atherosclerosis and endothelial dysfunction. For those with more severe and/or prolonged disease, there should be a specific focus on screening for cardiovascular disease risk (7). The correlation between psoriasis and atherosclerotic plaques involves similar inflammatory pathways related to T helper cells, impaired angiogenesis, and endothelial dysfunction (8). The overall relationship between LE8 scores and the eight factors covered by LE8 and psoriasis requires further investigation.

This cross-sectional study used data from the National Health and Nutrition Examination Survey (NHANES) to examine the potential relationship between LE8 and psoriasis among the US adult population, offering new strategies for the prevention and management of psoriasis in clinical practice.

## 2 Method

The study employed data from the National Health and Nutrition Examination Survey conducted between 2003–2006 and 2009–2014. Multivariable logistic regression and restricted cubic spline models were utilized to assess the relationships, revealing linear dose–response relationships. Subgroup analyses based on age, sex, poverty income ratio, education levels, and marital status demonstrated inverse associations between LE8 and psoriasis. All data are publicly accessible and can be obtained from the NHANES website.^1^

### 2.1 Study population

NHANES aims to assess the prevalence of primary illnesses and disease-specific risk factors in the United States, and a comprehensive overview of the survey can be found at http://www.cdc.gov/nchs/nhanes.htm. The survey employed sophisticated multiperiod probability-based sampling methods to obtain nationally representative samples. The NCHS Institutional Review Board approved the NHANES research protocol, and participants provided written informed consent at the time of enrollment.^2^ As this study is based on publicly available de-identified data, no ethical approval or consent is required.

**Figure 1:**
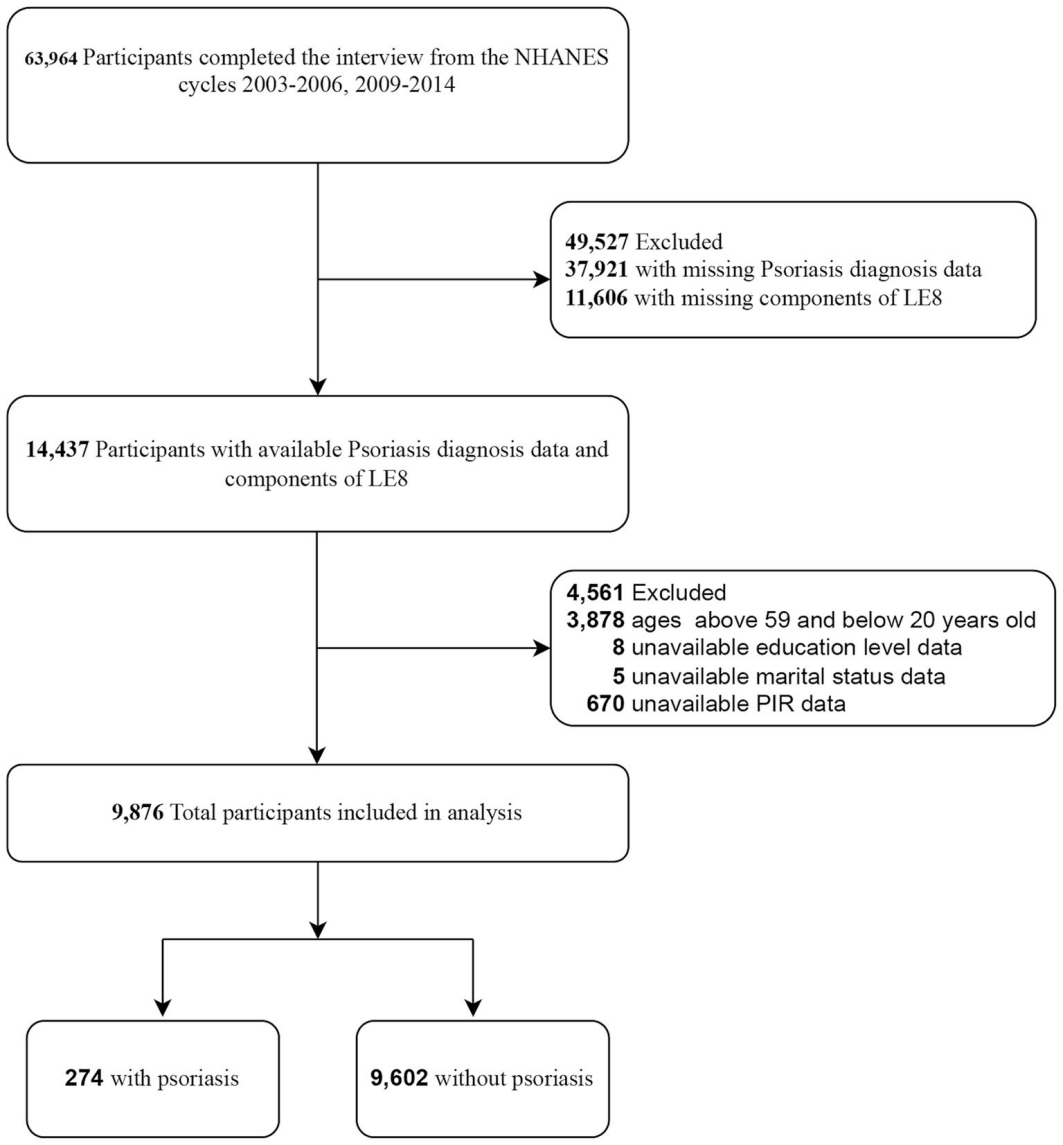
Selection of participants in the study. LE8, Life’s Essential 8; NHANES, national health and nutrition examination survey. PIR, family poverty income ratio.

This cross-sectional study utilized NHANES data from 2003 to 2006 and 2009 to 2014, adhering to the standards for Strengthening the Reporting of Observational Studies in Epidemiology. The investigation focused on adults aged 20 years and older with available LE8 score and psoriasis data. Exclusion criteria included missing demographic data (including educational level and marital status) (*n* = 13) and unavailable hypertension data (*n* = 11). Ultimately, 9,876 participants aged 20 years and older were included from a total of 26,043 participants in the 2003–2006 and 2009–2014 NHANES cycles. The flowchart for participant enrollment is depicted in Figure 1.

### 2.2 Measurements of LE8

The American Heart Association recently updated Life’s Simple 7 to LE8 in order to quantify cardiovascular health (CVH), encompassing 4 health behaviors and 4 health factors. This enhancement aims to provide more effective guidance for improving CVH in the general population. The detailed calculation of scores for each LE8 metric using NHANES data can be found in Supplementary Table S1. Each of the LE8 indicators was rated on a scale of 0 to 100, and the unweighted mean of these 8 indicators was used to calculate the total LE8 score. The American Heart Association suggested that participants with LE8 scores ≥80 be classified as having high CVH, those with scores of 50 to 79 as having moderate CVH, and those with scores <50 as having low CVH (3).

The Healthy Eating Index–2015 was employed to assess the diet metric. Supplementary Table S2 outlines the components and criteria for scoring the Healthy Eating Index–2015. The Healthy Eating Index–2015 score was calculated using information from the first 24-h diet recall interview conducted during the NHANES mobile examination center visit. If two 24-h recalls were available, the first one providing diet data was used. Information on physical activity, nicotine exposure, sleep health, diabetes, and medication history was obtained through self-report questionnaires. During the physical examination, participants’ blood pressure, height, and weight were professionally measured. Blood samples were collected for analysis of blood lipids, plasma glucose, and glycated hemoglobin at central laboratories.

Health behaviors encompass four factors: diet, physical activity, nicotine exposure, and sleep duration. The health behaviors score is the total score of these four factors divided by 4.

Health factors include four metrics: body mass index (BMI), non-high-density-lipoprotein cholesterol, blood glucose, and blood pressure. The health factors score is the total score of these four metrics divided by 4.

### 2.3 Diagnosis of psoriasis

Psoriasis was defined as an affirmative response to the question, “Have you ever been told by a health care provider that you had psoriasis?”

### 2.4 Covariates

Based on the literature, the following covariates were considered: age, sex, race and ethnicity, educational level, family income, marital status, diabetes, smoking status, alcohol drinking status, fasting total cholesterol, HDL cholesterol, diabetes history, hypertension history, physical activity, and sleep duration. In NHANES, self-reported race and ethnicity information were obtained from responses to survey questions on race and Hispanic ethnicity. As per NHANES categorization, participants were grouped into the following 4 races and ethnicities: non-Hispanic White, non-Hispanic Black, Mexican American, and Other (including non-Hispanic Black and multiracial). Educational level was classified into 3 levels (high school or less, some college, and college graduate or higher). For reporting NHANES dietary and health data, family income was categorized into 3 levels based on the family poverty income ratio: low income (≤1.3), medium income (>1.3 to 3.5), and high income (>3.5). Marital status was divided into 2 groups: married or living with partners and living alone. Diabetes history was determined by the survey question, “Has a doctor told you that you have diabetes?” Participants who answered “yes” were classified as having diabetes. Hypertension history was established by the survey question, “Have you ever been told that you have high blood pressure?” Participants who answered “yes” were classified as having hypertension. Smoking status was categorized into 3 groups: never smoker (or smoked <100 cigarettes), former smoker (smoked at least 100 cigarettes but has quit), and current smoker. Alcohol drinking status was determined by the survey question, “In any 1 year, have you had at least 12 drinks of any type of alcoholic beverage?” Participants who answered “yes” were classified as alcohol drinkers, while those who answered “no” were classified as non-alcohol drinkers.

### 2.5 Statistical analysis

We conducted a descriptive analysis for all participants. Categorical variables were presented as numbers and percentages, while continuous variables were expressed as mean and standard deviation (SD) for normally distributed data, or median and interquartile range for skewed distributions. We employed the chi-square test, *T*-test, and Kruskal-Wallis test to compare categorical variables, normally distributed continuous variables, and non-normally distributed continuous variables, respectively.

The correlation between covariates and psoriasis status was explored using univariate multinomial logistic regression. To assess the relationship between dietary LE8, health behaviors score, health factors score, and psoriasis, we utilized multivariate multinomial logistic regression models. We calculated odds ratios (ORs) and 95% confidence intervals (CIs). We established three models: the crude model did not adjust any confounders; Model 1 was adjusted for sociodemographic factors such as age (as a continuous variable), sex, and race/ethnicity; Model 2 was the fully adjusted model, which included adjustments for all factors in Model 1 plus poverty ratio (as a continuous variable), education levels, and marital status. To confirm the findings of LE8 score, health behaviors score, and health factors score as continuous variables and to test for nonlinearity, we categorized the above three scores according to the literature and calculated the p for trend (9).

To further explore nonlinearity, we conducted restricted cubic spline (RCS) regression with the continuous variables of LE8 score, health behaviors score, and health factors score to examine the dose–response relationship between these scores and psoriasis (7).

Furthermore, we investigated the associations between LE8 and psoriasis in different populations through subgroup analysis based on age strata, sex, PIR levels, education levels, and marital status. The significance of interactions was assessed using *p* values for the interaction coefficients between LE8 and subgroup populations.

All analyses were performed using the statistical software packages R 3.3.2 (http://www.R-project.org, The R Foundation) and Free Statistics software versions 1.9.2 (10). We conducted two-tailed tests, and *p* < 0.05 was considered statistically significant.

## 3 Results

### 3.1 Population characteristics

The study involved 9,876 participants, with an average (SD) age of 39.3 (11.3) years, of whom 5,084 (51.5%) were female. Among the total population, 44.6% were non-Hispanic White, 20.4% were non-Hispanic Black, and 16.0% were Mexican American; 59.1% had a college degree or higher. The percentage of married or cohabiting individuals was 61.0%, which is higher than those living alone. Table 1 presents the characteristics of the participants categorized by CVH groups. The prevalence of psoriasis was found to be 2.8% (95% CI, 5.26–6.56%). Participants in the high CVH group were typically younger, female, with higher income, college graduates or with higher education, and non-Hispanic white. They had a slim figure, drank alcohol, never smoked, had low levels of total cholesterol, high levels of HDL cholesterol, and no history of diabetes or hypertension. They spent significantly less time engaging in physical activity each day and considerably more time sleeping, as indicated in Table 1 (unweighted data used to describe the baseline characteristics of the patients).

**Table 1:** Baseline characteristics by CVH score level in the NHANES 2003–2006 and 2009–2014 cycles.

Variables	Total (*n* = 9,876)	Low CVH (*n* = 986)	Moderate CVH (*n* = 6,493)	High CVH (*n* = 2,397)	*p*
Psoriasis, *n* (%)					0.002
Without	9,602 (97.2)	944 (95.7)	6,310 (97.2)	2,348 (98)	
With	274 (2.8)	42 (4.3)	183 (2.8)	49 (2)	
Gender, *n* (%)					< 0.001
Male	4,792 (48.5)	455 (46.1)	3,414 (52.6)	923 (38.5)	
Female	5,084 (51.5)	531 (53.9)	3,079 (47.4)	1,474 (61.5)	
Age, mean ± SD	39.3 ± 11.3	44.4 ± 9.9	39.9 ± 11.2	35.5 ± 10.9	< 0.001
Race/ethnicity, *n* (%)					< 0.001
Non-Hispannic White	4,407 (44.6)	466 (47.3)	2,826 (43.5)	1,115 (46.5)	
Non-Hispannic Black	2018 (20.4)	272 (27.6)	1,460 (22.5)	286 (11.9)	
Mexican American	1,585 (16.0)	113 (11.5)	1,144 (17.6)	328 (13.7)	
Other race, including multiracial	1866 (18.9)	135 (13.7)	1,063 (16.4)	668 (27.9)	
Education, *n* (%)					< 0.001
High school or less	622 (6.3)	84 (8.5)	436 (6.7)	102 (4.3)	
College	3,421 (34.6)	507 (51.4)	2,473 (38.1)	441 (18.4)	
College graduate or higher	5,833 (59.1)	395 (40.1)	3,584 (55.2)	1854 (77.3)	
Marital status, *n* (%)					0.003
Married or living with partners	6,026 (61.0)	561 (56.9)	4,032 (62.1)	1,433 (59.8)	
Living alone	3,850 (39.0)	425 (43.1)	2,461 (37.9)	964 (40.2)	
PIR, *n* (%)					< 0.001
Low income	3,184 (32.2)	459 (46.6)	2,154 (33.2)	571 (23.8)	
Medium income	3,382 (34.2)	351 (35.6)	2,275 (35)	756 (31.5)	
High income	3,310 (33.5)	176 (17.8)	2064 (31.8)	1,070 (44.6)	
BMI, kg/m^2^ *n* (%)					< 0.001
<25.0	3,088 (31.3)	50 (5.1)	1,532 (23.6)	1,506 (62.8)	
25.0–29.9	3,136 (31.8)	164 (16.6)	2,245 (34.6)	727 (30.3)	
30.0–34.9	1993 (20.2)	309 (31.3)	1,554 (23.9)	130 (5.4)	
35.0–39.9	933 (9.4)	209 (21.2)	702 (10.8)	22 (0.9)	
> = 40.0	726 (7.4)	254 (25.8)	460 (7.1)	12 (0.5)	
Alcohol drinking status, *n* (%)					< 0.001
Yes	7,134 (76.4)	725 (77.5)	4,761 (77.4)	1,648 (73.3)	
No	2,203 (23.6)	211 (22.5)	1,391 (22.6)	601 (26.7)	
Smoking status, *n* (%)					< 0.001
Never	4,198 (57.5)	167 (23)	2,607 (53.3)	1,424 (84.8)	
Former	1,326 (18.2)	137 (18.8)	969 (19.8)	220 (13.1)	
Current	1778 (24.3)	423 (58.2)	1,319 (26.9)	36 (2.1)	
Fast total cholesterol(mg/dL), Mean ± SD	193.4 ± 40.5	217.3 ± 48.4	196.0 ± 39.7	176.8 ± 31.5	< 0.001
HDL Cholesterol(mg/dL), Mean ± SD	52.2 ± 15.4	45.9 ± 13.6	50.9 ± 14.8	58.6 ± 15.5	< 0.001
Diabetes, *n* (%)					< 0.001
Yes	668 (6.8)	272 (27.6)	380 (5.9)	16 (0.7)	
No	9,075 (91.9)	687 (69.7)	6,022 (92.7)	2,366 (98.7)	
Borderline	133 (1.3)	27 (2.7)	91 (1.4)	15 (0.6)	
Hypertension, *n* (%)					< 0.001
Yes	2,271 (23.0)	532 (54)	1,577 (24.3)	162 (6.8)	
No	7,594 (76.9)	453 (45.9)	4,911 (75.6)	2,230 (93)	
Physical activity, minites per day, Mean ± SD	11 (0.1)	1 (0.1)	5 (0.1)	5 (0.2)	
Sleep duration(hour), Mean ± SD	6.8 ± 1.4	6.0 ± 1.8	6.7 ± 1.4	7.2 ± 1.0	< 0.001
LE8 total score, Mean ± SD	68.7 ± 14.4	41.8 ± 6.5	66.1 ± 8.0	86.8 ± 5.1	< 0.001
Physical activity score, Median (IQR)	30.0 (20.0, 60.0)	30.0 (15.0, 62.5)	30.0 (20.0, 60.0)	30.0 (20.0, 60.0)	0.761
HEI-2015 diet score, Median (IQR)	25.0 (0.0, 50.0)	25.0 (0.0, 25.0)	25.0 (0.0, 50.0)	50.0 (25.0, 80.0)	< 0.001
Physical activity score,Median (IQR)	100.0 (40.0, 100.0)	0.0 (0.0, 90.0)	100.0 (40.0, 100.0)	100.0 (100.0, 100.0)	< 0.001
Nicotine exposure score, Median (IQR)	100.0 (25.0, 100.0)	0.0 (0.0, 75.0)	100.0 (0.0, 100.0)	100.0 (100.0, 100.0)	< 0.001
Body mass index score, Median (IQR)	70.0 (30.0, 100.0)	30.0 (0.0, 30.0)	70.0 (30.0, 70.0)	100.0 (70.0, 100.0)	< 0.001
Blood glucose score, Mean ± SD	89.2 ± 22.2	65.8 ± 32.0	89.3 ± 21.2	98.4 ± 8.6	< 0.001
Blood pressure score, Mean ± SD	76.8 ± 27.8	52.2 ± 30.1	74.6 ± 27.5	93.0 ± 15.4	< 0.001
Blood lipids score, Mean ± SD	66.0 ± 30.9	41.0 ± 28.6	62.7 ± 30.0	85.2 ± 22.8	< 0.001
NHANES cycles, *n* (%)					< 0.001
2005–2006	2,247 (21.3)	238 (22.8)	1,651 (23.8)	358 (14.1)	
2009–2010	2,940 (27.9)	284 (27.2)	1,943 (26)	713 (28)	
2011–2012	2,576 (24.5)	252 (24.1)	1,624 (23.4)	700 (27.5)	
2013–2014	2,772 (26.3)	272 (26)	1,729 (24.9)	771 (30.3)	

n, number; PIR, family poverty income ratio; LE8, Life’s Essential 8; CVH, cardiovascular diseases.

### 3.2 Association between components of LE8, health behaviors, health factors and psoriasis

Table 2 displays the association between each component of LE8, health behaviors, and health factors, and psoriasis in univariate logistic regression. The findings suggest that a high health factors score, particularly lower BMI (as indicated by the elevated body mass index score within the LE8 component), might have potential benefits in lowering the risk of psoriasis. The other scores do not demonstrate statistical significance.

**Table 2:** Associations between components of LE8, health behaviors, health factors and psoriasis.

Variable	OR(95%CI)	*P* value
HEI-2015 diet score		
Low (0–49)	1 (Reference)	
Moderate (50–79)	1.120 (0.842 ~ 1.489)	0.435
High (80–100)	0.907 (0.661 ~ 1.244)	0.545
Physical activity score		
Low (0–49)	1 (Reference)	
Moderate (50–79)	0.997 (0.558 ~ 1.783)	0.992
High (80–100)	1.137 (0.862 ~ 1.502)	0.363
Nicotine exposure score		
Low (0–49)	1 (Reference)	
Moderate (50–79)	1.263 (0.903 ~ 1.766)	0.172
High (80–100)	0.680 (0.515 ~ 0.898)	0.007
Sleep health score		
Low (0–49)	1 (Reference)	
Moderate (50–79)	1.200 (0.825 ~ 1.745)	0.341
High (80–100)	1.065 (0.761 ~ 1.491)	0.713
Health behaviors score		
Low (0–49)	1 (Reference)	
Moderate (50–79)	0.961 (0.713 ~ 1.295)	0.791
High (80–100)	0.892 (0.632 ~ 1.257)	0.513
Body mass index score		
Low (0–49)	1 (Reference)	
Moderate (50–79)	0.767 (0.577 ~ 1.02)	0.068
High (80–100)	0.681 (0.506 ~ 0.916)	0.011
Blood lipids score		
Low (0–49)	1 (Reference)	
Moderate (50–79)	0.830 (0.604 ~ 1.142)	0.253
High (80–100)	0.902 (0.684 ~ 1.19)	0.465
Blood glucose score		
Low (0–49)	1 (Reference)	
Moderate (50–79)	1.080 (0.65 ~ 1.793)	0.767
High (80–100)	0.779 (0.498 ~ 1.218)	0.273
Blood pressure score		
Low (0–49)	1 (Reference)	
Moderate (50–79)	0.790 (0.544 ~ 1.147)	0.215
High (80–100)	0.727 (0.512 ~ 1.031)	0.074
Health factors score		
Low (0–49)	1 (Reference)	
Moderate (50–79)	0.814 (0.573 ~ 1.157)	0.251
High (80–100)	0.606 (0.419 ~ 0.878)	0.008

HEI, Healthy Eating Index; LE8, Life’s Essential 8; and OR, odds ratio.

### 3.3 Association between LE8 score, health behaviors score, health factors score and psoriasis

**Figure 2:**
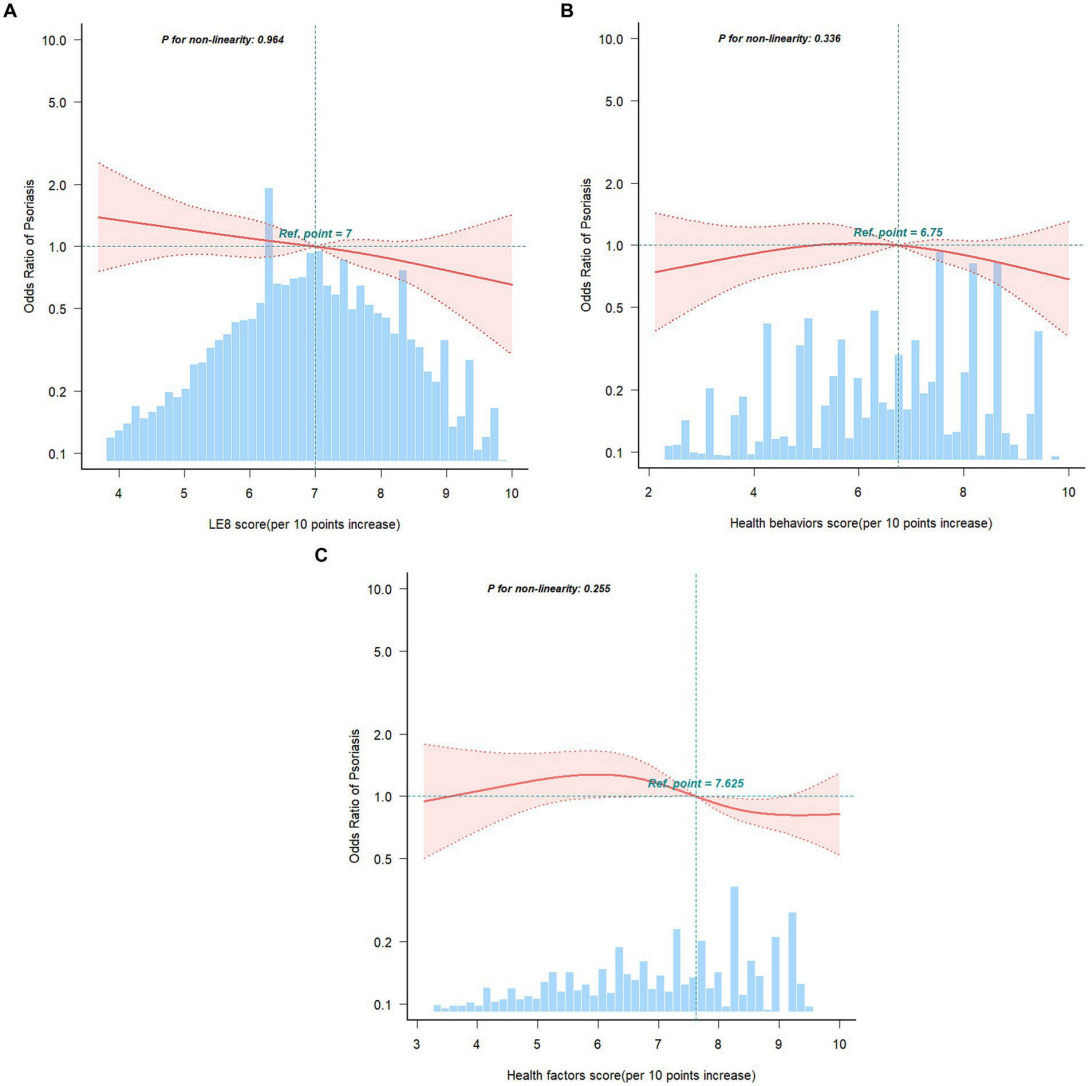
Restricted cubic spline fitting for the association between LE8 score **(A)**, health behaviors score **(B)**, health factors score **(C)** with psoriasis. Adjusted for age (as a continuous variable), sex, race/ethnicity, poverty ratio (as a continuous variable), education levels, and marital status. The red line and pink area in distribution histograms represent the estimated values and their corresponding 95% confidence intervals, respectively.

Multivariable logistic regression models and smooth curve fitting were employed to investigate the connections between LE8 score, health behaviors score, health factors score, and psoriasis. We observed that the relationship between LE8 score, health behaviors score, health factors score, and risk of psoriasis was linear and negative (Figure 2). In the two-piecewise regression models, model 1 remained unadjusted for any covariates, while model 2 included adjustments for age (as a continuous variable), sex, and race/ethnicity. Model 3 underwent further adjustments for poverty ratio (as a continuous variable), education levels, and marital status. Table 3 delineated the associations between LE8 score, health behaviors score, health factors score, and psoriasis. In the unadjusted model, each 10-point increase in the LE8 score was linked to a reduced prevalence of psoriasis (OR: 0.88, 95% CI: 0.82–0.96). Regarding the categorical variable, compared to the low CVH level, the moderate CVH level (OR: 0.65, 95% CI: 0.46–0.92) and high CVH level (OR: 0.47, 95% CI: 0.31–0.71) exhibited a lower prevalence of psoriasis in the unadjusted model. This association remained significant and independent of latent confounders. The trend for health factors score mirrored that of the LE8 score. However, there was no significant association observed between health behaviors score and psoriasis.

**Table 3:** Association of the Life’s Essential 8 scores, health behaviors score, health factors score with psoriasis.

Variable	Model 1		Model 2		Model 3	
	OR (95%CI)	*P* value	OR (95%CI)	*P* value	OR (95%CI)	*P* value
LE8 score						
Low (0–49)	1(Reference)		1(Reference)		1(Reference)	
Moderate (50–79)	0.65 (0.46 ~ 0.92)	0.014	0.72 (0.51 ~ 1.02)	0.062	0.73 (0.51 ~ 1.04)	0.078
High (80–100)	0.47 (0.31 ~ 0.71)	<0.001	0.50 (0.33 ~ 0.78)	0.002	0.51 (0.32 ~ 0.8)	0.003
p for trend		0.001		0.002		0.003
Per 10 points increase	0.88 (0.82 ~ 0.96)	0.003	0.90 (0.83 ~ 0.98)	0.013	0.90 (0.83 ~ 0.99)	0.027
Health behaviors score						
Low (0–49)	1(Reference)		1(Reference)		1(Reference)	
Moderate (50–79)	0.96 (0.71 ~ 1.29)	0.791	1 (0.74 ~ 1.36)	0.978	1.04 (0.76 ~ 1.41)	0.807
High (80–100)	0.89 (0.63 ~ 1.26)	0.513	0.88 (0.62 ~ 1.24)	0.462	0.94 (0.65 ~ 1.35)	0.721
p for trend		0.509		0.449		0.707
Per 10 points increase	0.96 (0.91 ~ 1.02)	0.202	0.96 (0.91 ~ 1.02)	0.200	0.97 (0.91 ~ 1.04)	0.369
Health factors score						
Low (0–49)	1(Reference)		1(Reference)		1(Reference)	
Moderate (50–79)	0.81 (0.57 ~ 1.16)	0.251	0.85 (0.59 ~ 1.21)	0.361	0.86 (0.6 ~ 1.24)	0.426
High (80–100)	0.61 (0.42 ~ 0.88)	0.008	0.65 (0.44 ~ 0.97)	0.034	0.67 (0.45 ~ 1)	0.047
p for trend		0.004		0.021		0.030
Per 10 points increase	0.90 (0.85 ~ 0.96)	0.001	0.92 (0.86 ~ 0.98)	0.010	0.92 (0.86 ~ 0.98)	0.015

OR, odds ratio. CI, confidence interval. LE8, life’s essential 8. Model 1, creude model, no covariables were adjusted. Model 2 Adjusted for age (as a continuous variable), sex, race/ethnicity. Model 3 Additionally adjusted for poverty ratio (as a continuous variable), education levels, and marital status.

### 3.4 Subgroup analysis

**Figure 3:**
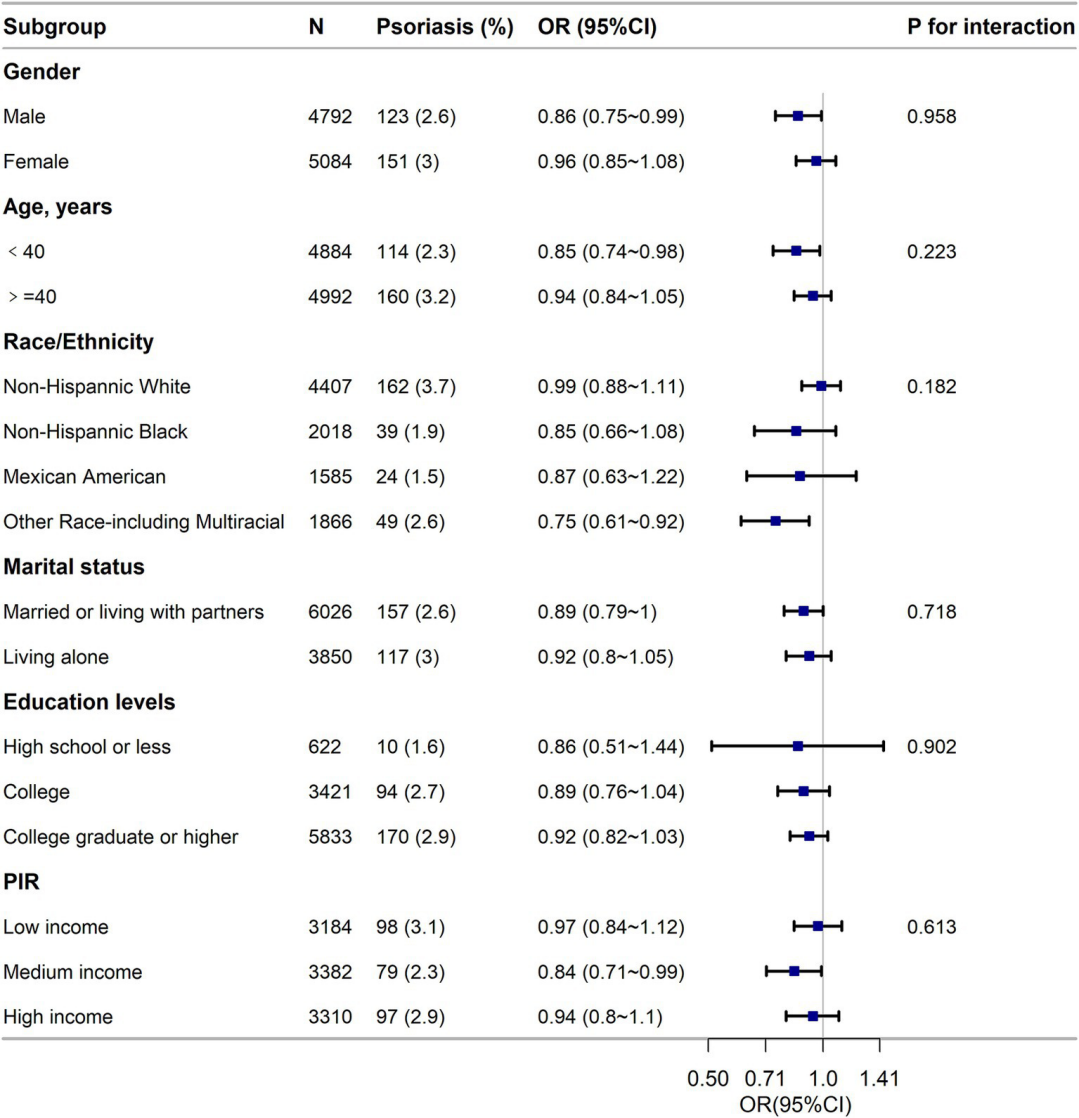
Subgroup analysis of the association of the Life’s Essential 8 scores and the risk of psoriasis. ORs were calculated as per score increase in LE8 total score. Each stratification was adjusted for gender, age (as a categorical variable), race/ethnicity, marital status, education level and family poverty income ratio (as a categorical variable). OR, odds ratio; CI, confidence interval; n, number; PIR, family poverty income ratio.

The results of the subgroup analysis in Figure 3 suggest a strong inverse relationship between LE8 components and psoriasis across different population subgroups, and the findings were dependable and in line with the initial results.

## 4 Discussion

Our findings not only corroborate the recent work by Wang et al. (2), which first demonstrated an association between lower LE8 scores and psoriasis in a similar population, but also substantially extend these insights in several key aspects.

This study demonstrates a significant relationship between higher LE8 scores and lower prevalence of psoriasis, as well as a notable association between higher nicotine exposure scores, BMI, and lower psoriasis risk. Subgroup and sensitivity analyses indicate that the negative correlation between LE8 and psoriasis aligns with the overall findings.

Psoriasis, a chronic and recurrent immune-mediated inflammatory skin disease, varies in prevalence by country, can occur at any age (11), and is characterized by genetic susceptibility (12), adversely impacting the physical, social, psychological, and emotional well-being of patients (13). According to the methodology of the Global Burden of Disease Study 2019, there were an estimated 4,622,594 new cases of psoriasis worldwide in 2019. The age-standardized incidence rate in 2019 was 57.8 per 100,000 people (14). It represents a significant public health concern (15).

The typical skin manifestations of psoriasis include localized or widespread scaly erythematous plaques, with the removal of adherent scales resulting in pinpoint bleeding. Biologic therapies have become a pivotal point in the management of the disease, offering an effective and reasonably safe alternative for patients inadequately controlled by conventional therapies (16). Psoriasis is mediated by Th17 and Th23, leading to abnormal production of inflammatory mediators and excessive proliferation of keratinocytes. While Th-1 cells have long been a hallmark of psoriasis management (17), recent research has highlighted the prominence of Th17 cells, which are present in the dermis of psoriatic lesions and mediate skin inflammation upon recognition of self-lipid antigens presented by CD1a (18). The IL-23/Th-17 axis plays a crucial role in the exacerbation and treatment of psoriasis. In cardiovascular diseases (19), Th17 cells play a key role in thrombosis and atherosclerosis (20).

Previous studies have demonstrated that psoriasis patients exhibit higher cardiovascular prevalence factors, including metabolic syndrome, diabetes, hypertension, smoking, obesity, insulin resistance, and dyslipidemia (21–24). Research by Snekvik et al. (25) suggests a positive correlation between psoriasis and obesity indices and cardiovascular risk factors, particularly significant in moderate to severe psoriasis patients. Alexandre et al. (26) found that psoriasis patients exhibit lower levels of physical activity due to psychological and physiological reasons, potentially increasing their risk of cardiovascular diseases. Dyslipidemia, also a cardiovascular risk factor, is associated with psoriasis, although Ma’s et al. (27) research found no significant correlation between psoriasis and certain lipid level changes. Additionally, sleep deprivation is common among psoriasis patients, linked to psychological and physiological factors (28). Studies on smoking habits have revealed the following: one study found that psoriasis patients have a higher smoking rate compared to the control group, which may directly lead to or exacerbate chronic obstructive pulmonary disease, resulting in subclinical airway inflammation in patients (29). Another study (30, 31) found that psoriasis patients have underlying airway inflammation, so they tend to smoke more. Despite a significant global decrease in smoking rates from 1990 to 2019 (decreasing by 1.21% annually), the use of smokeless tobacco has not improved (actually increasing by 0.46% annually).

The meta-analysis also revealed a close association between psoriasis and cardiovascular disease. Liu et al.’s (32) study demonstrated the association of psoriasis with all adverse cardiovascular outcomes, particularly in severe patients, indicating psoriasis as an independent risk factor for adverse cardiovascular outcomes. Furthermore, Garshick et al. (33) indicated that patients with psoriasis have over a 50% increased risk of developing cardiovascular disease, with this cardiovascular risk increasing with the severity of skin involvement. Zhang et al.’s (34) research, combining Mendelian randomization, provided preliminary evidence suggesting a shared genetic origin between psoriasis and CVD, and targeted psoriasis treatments may improve cardiovascular outcomes. Additionally Zhang et al. (34), Galajda et al. (35) found that early use of TNF inhibitors may help reduce the cardiovascular disease risk in psoriasis patients.

While the underlying mechanisms between LE8 and psoriasis remain elusive, extensive research has revealed the critical roles of metabolic syndrome and lifestyle in the development of psoriasis, both of which are potential health factors and indicators of healthy behaviors in LE8 (36–38).

In conclusion, our study results indicate that maintaining optimal cardiovascular health can reduce the prevalence of developing psoriasis. These findings provide important guidance for the management and prevention of psoriasis.

The strengths of this study include: First, the use of a large, nationally representative sample enhances the generalizability of our findings. Second, this is the first study to employ restricted cubic spline models to reveal a linear dose-response relationship between LE8 score and psoriasis, quantifying the risk reduction associated with each 10-point increase in LE8. Third, we systematically evaluated the association between individual LE8 components and psoriasis, identifying nicotine exposure and BMI as key drivers of this association. Fourth, comprehensive subgroup analyses validated the robustness of our findings across different populations based on gender, age, race/ethnicity, marital status, education level, and income level. Fifth, compared to previous research including the foundational work by Wang et al. (2), our analysis provides more precise effect estimates and demonstrates the consistency of the LE8-psoriasis association across diverse subgroups.

However, some limitations should be considered in this study. Firstly, despite controlling for several potential confounding variables, the cross-sectional nature of the study precludes establishing a causal relationship between LE8 and psoriasis prevalence. Further research is needed to explore the longitudinal causal relationship between LE8 and psoriasis prevalence. Secondly, the assessment of some indicators in the LE8 assessment section was based on questionnaire surveys, which may lead to estimation errors. Thirdly, the impact of non-random missing data on the results cannot be ruled out due to baseline differences between included and excluded individuals. Finally, the generalizability of the correlations identified in this study to younger individuals or populations in other countries remains uncertain and requires further investigation.

## 5 Conclusion

The LE8 score may be negatively associated with the prevalence of psoriasis in the adult population in the United States. Additionally, higher scores in the assessment of nicotine exposure and BMI should be prioritized within the LE8 components. These findings suggest that LE8, as a practical and beneficial composite indicator for improving cardiovascular health (CVH), can be applied in clinical practice to aid in the early identification of psoriasis prevalence among patients and the general population, thereby minimizing the burden of psoriasis. Dermatologists should provide appropriate non-pharmacological advice to patients, including smoking cessation and weight management.

## Data Availability

Publicly available datasets were analyzed in this study. This data can be found at: https://www.cdc.gov/nchs/nhanes/index.htm.

## Footnotes

^1^https://www.cdc.gov/nchs/nhanes/index.htm

^2^https://www.cdc.gov/nchs/nhanes/irba98.htm
