# Correction: Association between Life's Essential 8 and psoriasis in US adults: a cross-sectional study

**DOI:** 10.3389/fmed.2026.1839887

**Published:** 2026-05-06

**Authors:** Junjie Zhang, Ci Ren, Zihan Qin, Ling Zhu, Zhoufeng Jin, Yuanyuan Yan, Xinghe Pan, Lan Luan

**Affiliations:** 1 Department of Pathology, Central Hospital Affiliated to Shenyang Medical College, Shenyang, China; 2 Department of Dermatological Surgery, Shenyang Seventh People's Hospital, Shenyang, China; 3 Department of Gastroenterology, Beijing Friendship Hospital, Capital Medical University, Beijing, China; 4 State Key Laboratory of Digestive Health, Beijing, China; 5 National Clinical Research Center for Digestive Diseases, Beijing, China; 6 Medical Technology Department, Sichuan Nursing Vocational College, Chengdu, China; 7 Department of Plastic Surgery, Shenyang Mingliu Plastic Surgery and Aesthetics Hospital, Shenyang, China; 8 Affiliated Hospital of Tianjin Academy of Traditional Chinese Medicine, Tianjin, China; 9 Department of General Surgery, Central Hospital Affiliated to Shenyang Medical College, Shenyang, China

**Keywords:** psoriasis, Life's Essential 8, cardiovascular health, NHANES, risk factors

Wang et al. (2023) was not cited in the article. The citation has now been inserted in the section [Introduction (Page 2, Line 54–57) and Discussion (Page 7, Lines 239–241; Page 8, 291–293)] and should read:

“[Psoriasis, a chronic immune-mediated systemic disease, is characterized by red patches covered with silvery scales, often causing itching, pain, and in severe cases, joint involvement. It is associated with cardiovascular diseases, diabetes, and metabolic syndrome, significantly impacting patients' quality of life. Early identification and treatment of cardiovascular complications are crucial for improving patient outcomes (1). A recent study utilizing NHANES data from 2005–2014 first applied the Life's Essential 8 (LE8) metric to the psoriasis population and found that individuals with psoriasis had a higher prevalence of poor cardiovascular health (CVH), as indicated by lower LE8 scores, compared to those without psoriasis (2). This pioneering work highlighted the utility of LE8 in this high-risk population. However, a comprehensive dose-response analysis and investigation of the independent contributions of individual LE8 components to psoriasis risk remain unexplored.

Our findings not only corroborate the recent work by Wang et al. (2), which first demonstrated an association between lower LE8 scores and psoriasis in a similar population, but also substantially extend these insights in several key aspects.

The strengths of this study include: First, the use of a large, nationally representative sample enhances the generalizability of our findings. Second, this is the first study to employ restricted cubic spline models to reveal a linear dose-response relationship between LE8 score and psoriasis, quantifying the risk reduction associated with each 10-point increase in LE8. Third, we systematically evaluated the association between individual LE8 components and psoriasis, identifying nicotine exposure and BMI as key drivers of this association. Fourth, comprehensive subgroup analyses validated the robustness of our findings across different populations based on gender, age, race/ethnicity, marital status, education level, and income level. Fifth, compared to previous research including the foundational work by Wang et al. (2), our analysis provides more precise effect estimates and demonstrates the consistency of the LE8-psoriasis association across diverse subgroups.]”

The original version of this article has been updated.
