# Supplementary material for: Association between Life’s Essential 8 and psoriasis in US adults: a cross-sectional study
Source: Front Med (Lausanne). 2024 Oct 10;11:1445288. doi: 10.3389/fmed.2024.1445288 (PMC11499175; doi:10.3389/fmed.2024.1445288)
Supplement: Supplementary file 1 [file Table_1.DOCX]

**Supplementary Table 1. Scoring Criteria for the DASH-style diet score.**

| **Domain** | **CVH metric** | **Method of measurement** | **Quantification of CVH metric** | **Score, points** |
| --- | --- | --- | --- | --- |
| Health Behaviors | Diet | Quantiles of HEI-2015 (population)  Example tools: 24-hour dietary recall | 1st–24th | 0 |
|  |  |  | 25th–49th | 25 |
|  |  |  | 50th–74th | 50 |
|  |  |  | 75th–94th | 80 |
|  |  |  | ≥95th | 100 |
|  | PA | Self-reported minutes of moderate or vigorous PA per week  Example tools: NHANES PAQ | 0 | 0 |
|  |  |  | 1–29 | 20 |
|  |  |  | 30–59 | 40 |
|  |  |  | 60–89 | 60 |
|  |  |  | 90–119 | 80 |
|  |  |  | 120–149 | 90 |
|  |  |  | ≥150 | 100 |
|  | Nicotine  exposure | Self-reported use of cigarettes or inhaled  NDS  Example tools: NHANES SMQ and SMQFAM | Current smoker | 0 |
|  |  |  | Former smoker quit <1 y, or currently using inhaled NDS, with active indoor smoker | 5 |
|  |  |  | Former smoker, quit <1 y, or currently using inhaled NDS, without active indoor smoker | 25 |
|  |  |  | Former smoker, quit 1–<5 y, with active indoor smoker | 30 |
|  |  |  | Former smoker, quit 1–<5 y, without active indoor smoker | 50 |
|  |  |  | Former smoker, quit ≥5 y, with active indoor smoker | 55 |
|  |  |  | Former smoker, quit ≥5 y, without active indoor smoker | 75 |
|  |  |  | Never smoker, with active indoor smoker | 80 |
|  |  |  | Never smoker, without active indoor smoker | 100 |
|  | Sleep health | Self-reported average hours of sleep per night  Example tools: NHANES SLQ | <4 | 0 |
|  |  |  | 4–<5 | 20 |
|  |  |  | 5–<6 or ≥10 | 40 |
|  |  |  | 6–<7 | 70 |
|  |  |  | 9–<10 | 90 |
|  |  |  | 7–<9 | 100 |
| Health factors | BMI | Body weight (kilograms) divided by height squared (meters squared) Example tools: NHANES BMX | ≥40.0 | 0 |
|  |  |  | 35.0–39.9 | 15 |
|  |  |  | 30.0–34.9 | 30 |
|  |  |  | 25.0–29.9 | 70 |
|  |  |  | <25 | 100 |
|  | Blood lipids | Plasma total and HDL cholesterol with calculation of non–HDL cholesterol Example tools: NHANES TCHOL, HDL and BPQ | ≥220 or 190–219 (take medication) | 0 |
|  |  |  | 190–219(no medication) or 160–189 (take medication) | 20 |
|  |  |  | 160–189(no medication) or 130–159(take medication) | 40 |
|  |  |  | 130–159(no medication) | 60 |
|  |  |  | <130(take medication) | 80 |
|  |  |  | <130(no medication) | 100 |
|  | Blood glucose | Casual HbA1c (%) | Diabetes with HbA1c ≥10.0 | 0 |
|  |  |  | Diabetes with Hb A1c 9.0–9.9 | 10 |
|  |  |  | Diabetes with HbA1c 8.0–8.9 | 20 |
|  | Blood glucose | Casual HbA1c (%)  Example tools: NHANES GHB and DIQ | Diabetes with HbA1c 7.0–7.9 | 30 |
|  |  |  | Diabetes with HbA1c <7.0 | 40 |
|  |  |  | No diabetes and HbA1c 5.7–6.4) | 60 |
|  |  |  | No history of diabetes and HbA1c <5.7 | 100 |
|  | BP | Appropriately measured systolic and diastolic BPs  Example tools: NHANES BPX and BPQ | ≥160 or ≥100 | 0 |
|  |  |  | 140–159 or 90–99(take medication) | 5 |
|  |  |  | 140–159 or 90–99(no medication) | 25 |
|  |  |  | 130–139 or 80–89(take medication) | 30 |
|  |  |  | 130–139 or 80–89(no medication) | 50 |
|  |  |  | 120–129/<80 (take medication) | 55 |
|  |  |  | 120–129/<80 (no medication) | 75 |
|  |  |  | <120/<80(take medication) | 80 |
|  |  |  | <120/<80(no medication) | 100 |
