# Supplementary material for: Association between Life’s Essential 8 and psoriasis in US adults: a cross-sectional study
Source: Front Med (Lausanne). 2024 Oct 10;11:1445288. doi: 10.3389/fmed.2024.1445288 (PMC11499175; doi:10.3389/fmed.2024.1445288)
Supplement: Supplementary file 2 [file Table_2.DOCX]

**Supplementary Table 2. Scoring Criteria for the DASH-style diet score.**

| **Component** | **Foods (NHANES 24-hour recall)** | **Scoring Criteria** | **Note** |
| --- | --- | --- | --- |
| Fruits | All fruits and fruit juices | Quintile 1: 1 point  Quintile 2: 2 points  Quintile 3: 3 points  Quintile 4: 4 points  Quintile 5: 5 points | Higher score represents more ideal intake  Quintile 1 is lowest  consumption and  Quintile 5 is highest  consumption |
| Vegetables | All vegetables except potatoes and legumes |  |  |
| Nuts and Legumes | Nuts and peanut butter, dried beans, peas, tofu |  |  |
| Whole Grains | Brown rice, dark breads, cooked cereal, whole grain cereal, other grains, popcorn, wheat germ, bran |  |  |
| Low-fat Dairy | Skim milk, yogurt, cottage cheese |  |  |
| Sodium | Sum of sodium content of all foods reported as consumed | Quintile 1: 5 points  Quintile 2: 4 points  Quintile 3: 3 points  Quintile 4: 2 points  Quintile 5: 1 point | Reverse scoring as  higher quintiles represent less ideal  intake  Quintile 1 is lowest  consumption and  Quintile 5 is highest  consumption |
| Red and Processed Meats | Beef, pork, lamb, deli meats, organ meats, hot dogs, bacon |  |  |
| Sweetened beverages | Carbonated and noncarbonated sweetened beverages |  |  |
